# Formulæ for Druggists

**Published:** 1871-07

**Authors:** 


### Formulæ for Druggists.

We present our Druggist subscribers with a valuable and reliable collection of formulæ for preparing Syrups and Mineral Waters, to be used in their Soda Fountains. They are taken from the *Druggists' Circular*, and are the very best in use.

### SYRUPS FOR SODA FOUNTAINS.

1. SIMPLE SYRUP.

White sugar................10 pounds.
Water..................... 1 gallon.
Isinglass (best)..............¼ ounce.
(Or, the white of an egg.) "

Dissolve the isinglass in hot water, and add it to the hot syrup. The syrup is to be made with gentle heat, and then strained.

2. LEMON SYRUP.

Grate off the yellow rind of lemons, and beat it up with a sufficient quantity of granulated sugar. Express the lemon-juice, add to each pint of juice 1 pint of water, and 3½ pounds of granulated sugar, including that rubbed up with the rind; warm until the sugar is dissolved, and strain.

3. ANOTHER FORMULA.

Simple syrup............... 1 gallon.
Oil of lemon...............25 drops.
Citrit acid.................10 drachms.

Rub the oil of lemon with the acid, add a small portion of syrup, and mix.

4. ANOTHER FORMULA.

Dissolve 6 drachms of tartaric acid and 1 ounce of gum-arabic, in pieces, in one gallon of simple syrup; then flavor with 1½ fluid drachms of best oil of lemon. Or, flavor with the saturated tincture of the peel in cologne spirits.

5. STRAWBERRY SYRUP.

Strawberry juice............1 pint.
Simple syrup...............3 pints.
Solution of citric acid (see below)....................2 drachms.

6. ANOTHER FORMULA.

Fresh strawberries.......... 5 quarts.
White sugar................12 pounds.
Water...................... 1 pint.

Sprinkle some of the sugar over the fruit in layers, and allow the whole to stand for several hours; express the juice and strain, washing out the pulp with water; add the remainder of sugar and water, bring the fluid to the point of boiling, and then strain. This will keep for a long time.

7. RASPBERRY SYRUP.

Raspberry juice............1 pint.
Simple syrup..............3 pints.
Solution of citric acid......2 drachms.

Raspberry syrup may also be made in a way similar to No. 6 for strawberry.

8. VANILLA SYRUP.

Fluid extract of vanilla...... 1 ounce.
Citric acid.................½ ounce.
Simple syrup............... 1 gallon.

Rub the acid with some of the syrup, add the extract of vanilla, and mix.

9. VANILLA CREAM SYRUP.

Fluid extract of vanilla......1 ounce.
Simple syrup...............3 pints.
Cream (or condensed milk)...1 pint.

May be colored with carmine.

10. CREAM SYRUP.

Fresh cream................½ pint.

Fresh milk.................½ pint.

Powdered sugar............ 1 pound.

Mix by shaking, and keep in a cool place. The addition of a few grains of bicarbonate of soda will for some time retard souring.

11. GINGER SYRUP.

Tincture of ginger......2 fl. ounces.
Simple syrup...........4 pints.

12. ORANGE SYRUP.

Oil of orange ..............30 drops.
Tartaric acid.............. 4 drachms.
Simple syrup............... 1 gallon.

Rub the oil with the acid, and mix.

13. PINEAPPLE SYRUP.

Oil of pineapple........... 1 drachm.
Tartaric acid............... 1 drachm.
Simple syrup............... 6 pints.

14. ANOTHER FORMULA.

Pineapple juice, by expression, 1 gallon.
Sugar.....................15 pounds.
Fruit acid (see below) ...... 2 ounces.

15. ORGEAT SYRUP.

Cream syrup ............... 1 pint.
Vanilla syrup .............. 1 pint.
Oil of bitter almonds........ 4 drops.

16. NECTAR SYRUP.

Vanilla syrup .............. 5 pints.
Pineapple syrup............ 1 pint.
Strawberry, raspberry or lemon 2 pints.

17. SHERBET SYRUP.

Vanilla syrup............. 3 pints.
Pineapple syrup............ 1 pint.
Lemon syrup .............. 1 pint.

18. GRAPE SYRUP.

Brandy.................... ½ pint.

Spirits of lemon........... ¼ ounce.

Tinct. of red sanders........ 2 ounces.

Simple syrup .............. 1 gallon.

19. BANANA SYRUP.

Oil of banana .............. 2 drachms.
Tartaric acid............... 1 drachm.
Simple syrup............... 6 pints.

20. COFFEE SYRUP.

Coffee, roasted ............ ½ pound.

Boiling water ............. 1 gallon.

Enough is filtered to make one-half gallon of the infusion, to which add

Granulated sugar........... 7 pounds.

21. WILD CHERRY SYRUP.

Wild cherry bark (in coarse powder) ................ 5 ounces.

Moisten the bark with water and let it stand for twenty-four hours in a close vessel. Then pack it firmly in a percolator, and pour water upon it until one pint of fluid is obtained. To this add

Sugar.....................28 ounces.

22. WINTER GREEN SYRUP.

Oil of wintergreen..........25 drops.
Simple syrup............... 5 pints.
Burnt sugar (to color) ...... q. s.

23. SARSAPARILLA SYRUP.

Oil of wintergreen ..........10 drops.
Oil of anise ...............10 drops.
Oil of sassafras............10 drops.
Fluid extract of sarsaparilla.. 2 ounces.
Simple syrup............... 5 pints.
Powdered extract of liquorice ½ ounce.

24. ANOTHER FORMULA. (Parrish's.)

Simple syrup............... 4 pints.
Comp. syrup of sarsaparilla.. 4 fl. ounces.
Caramel .................. 1½ ounces.
Oil of wintergreen .......... 6 drops.
Oil of sassafras ............ 6 drops.

25. MAPLE SYRUP.

| | |
|---|---|
| Maple sugar | 4 pounds. |
| Water | 2 pints. |

26. CHOCOLATE SYRUP.

| | |
|---|---|
| Best chocolate | 8 ounces. |
| Water | 2 pints. |
| White sugar | 4 pounds. |

Mix the chocolate in water, and stir thoroughly over a slow fire. Strain and add the sugar.

27. COFFEE CREAM SYRUP.

| | |
|---|---|
| Coffee syrup | 2 pints. |
| Cream | 1 pint. |

28. AMBROSIA SYRUP.

| | |
|---|---|
| Raspberry syrup | 2 pints. |
| Vanilla syrup | 2 pints. |
| Hock wine | 4 ounces. |

29. HOCK AND CLARET SYRUP.

| | |
|---|---|
| Hock or claret wine | 1 pint. |
| Simple syrup | 2 pints. |

30. SOLFERINO SYRUP.

| | |
|---|---|
| Brandy | 1 pint. |
| Simple syrup | 2 pints. |

31. FRUIT ACID (used in some of the syrups).

| | |
|---|---|
| Critic acid | 4 ounces. |
| Simple syrup | 8 ounces. |

Most of the syrups not made from fruits may have a little gum arabic added, in order to produce a rich froth.

### MINERAL WATERS.

### MINERAL WATERS FOR FOUNTAINS.

*Kissengen Water.*

| | |
|---|---|
| Bicarbonate of soda, | 1 drachm. |
| Carbonate of lime | 2 drachms, 2 scruples. |
| Precipitated carbonate of iron | 2 scruples. |
| Common salt | 8 ounces. |
| Muriate of ammonia | 4 grains. |
| Sulphate of soda | 2 drachms, 2 scruples. |
| Sulphate of magnesia | 2 ounces. |
| Phosphate of soda | 13 grains. |
| Phosphate of lime | 2 drac., 2 scru. |

Mix. Add water half gallon. Let it stand half a day, filter, add carbonate of magnesia, 3 drachms, 1 scruple, and charge with 10 gallons of water.

*Vichy Water.*

| | |
|---|---|
| Sulphate of potassa | 2 drachms. |
| Sulphate of soda | 4 scruples. |
| Phosphate of soda | 25 grains. |
| Common salt | 6 drachms. |
| Bicarbonate of soda | 5¼ ounces. |
| Carbonate of ammonia | 10 grains. |

Mix. Add water half gallon. Let it stand half a day, filter, and charge with 10 gallons.

*Congress Water.*

| | |
|---|---|
| Common salt | 7¾ ounces. |
| Hydrate of soda | 23 grains. |
| Bicarbonate of soda | 20 grains. |
| Calcined magnesia | 1 ounce. |

Add to 10 gallons of water and charge with gas.

